# Non-Coding RNAs and Endometrial Cancer

**DOI:** 10.3390/genes9040187

**Published:** 2018-03-29

**Authors:** Cristina Vallone, Giuliano Rigon, Caterina Gulia, Alberto Baffa, Raffaella Votino, Giulia Morosetti, Simona Zaami, Vito Briganti, Francesco Catania, Marco Gaffi, Roberto Nucciotti, Fabio Massimo Costantini, Roberto Piergentili, Lorenza Putignani, Fabrizio Signore

**Affiliations:** 1 Department of Obstetrics and Gynaecology, Misericordia Hospital Grosseto, Usl Sud-Est Toscana, 58100 Grosseto, Italy; cristina.vallone@uslsudest.toscana.it (C.V.); alberto.baffa@uslsudest.toscana.it (A.B.); raffaella.votino@uslsudest.toscana.it (R.V.); giuliamorosetti@gmail.com (G.M.); fabrizio.signore@uslsudest.toscana.it (F.S.); 2 Department of Obstetrics and Gynaecology, San Camillo-Forlanini Hospital, 00152 Rome, Italy; 3 Department of Pediatric Surgery and Urology, San Camillo-Forlanini Hospital, 00152 Rome, Italy; 85cate@live.it (C.G.); vito.briganti@fastwebnet.it (V.B.); 4 Unit of Forensic Toxicology (UoFT), Department of Anatomical Histological, Forensic and Orthopedic Sciences, Sapienza University of Rome, 00185 Roma, Italy; simona.zaami@uniroma1.it; 5 Department of Obstetrics and Gynaecology, Santa Maria alla Gruccia Hospital Valdarno, Usl Sud-Est Toscana, 52025 Montevarchi, Italy; simona.zaami@uniroma1.it; 6 Department of Urology, San Camillo-Forlanini Hospital, 00152 Rome, Italy; marco.gaffi59@gmail.com; 7 Department of Urology, Misericordia Hospital Grosseto, Usl Sud-Est Toscana, 58100 Grosseto, Italy; roberto.nucciotti@uslsudest.toscana.it (R.N.); roberto.nucciotti@uslsudest.toscana.it (F.M.C.); 8 Institute of Molecular Biology and Pathology, Italian National Research Council, 00185 Rome, Italy; roberto.piergentili@uniroma1.it; 9 Unit of Human Microbiome, Bambino Gesù Children’s Hospital, IRCCS, 00165 Rome, Italy; lorenza.putignani@opbg.net; 10 Unit of Parasitology, Bambino Gesù Children’s Hospital, IRCCS, 00165 Rome, Italy

**Keywords:** endometrial cancer, epigenetics, non-coding RNA (ncRNA), long non-coding RNA (lncRNA), small non-coding RNA (small ncRNA)

## Abstract

Non-coding RNAs (ncRNAs) are involved in the regulation of cell metabolism and neoplastic transformation. Recent studies have tried to clarify the significance of these information carriers in the genesis and progression of various cancers and their use as biomarkers for the disease; possible targets for the inhibition of growth and invasion by the neoplastic cells have been suggested. The significance of ncRNAs in lung cancer, bladder cancer, kidney cancer, and melanoma has been amply investigated with important results. Recently, the role of long non-coding RNAs (lncRNAs) has also been included in cancer studies. Studies on the relation between endometrial cancer (EC) and ncRNAs, such as small ncRNAs or micro RNAs (miRNAs), transfer RNAs (tRNAs), ribosomal RNAs (rRNAs), antisense RNAs (asRNAs), small nuclear RNAs (snRNAs), Piwi-interacting RNAs (piRNAs), small nucleolar RNAs (snoRNAs), competing endogenous RNAs (ceRNAs), lncRNAs, and long intergenic ncRNAs (lincRNAs) have been published. The recent literature produced in the last three years was extracted from PubMed by two independent readers, which was then selected for the possible relation between ncRNAs, oncogenesis in general, and EC in particular.

## 1. Introduction

The analysis of non-coding RNA (ncRNAs) expression in endometrial cancers (EC) might be of importance in understanding the neoplastic transformation of the endometrium. These RNA transcripts are possible biomarkers of early neoplastic transformation, but their significance might be even greater.

Approximately only 2% of the genome in humans has protein encoding functions, so most of the human genome is not translated into proteins. It has been called *genomic dark matter*, because no specific function has been described for it [1]. However, many information-carrying genes have been demonstrated to have significant regulatory functions, even if they remain untranslated. The ncRNAs include transfer RNAs (tRNAs), ribosomal RNAs (rRNAs), antisense RNAs (asRNA), micro RNAs (miRNAs), small nuclear RNAs (snRNAs), small nucleolar RNAs (snoRNAs), competing endogenous RNAs (ceRNAs) and piwi-interacting RNAs (piRNAs). 

Recently, an additional group of ncRNAs has been discovered: they are called long-ncRNAs (lncRNAs), as they are over 200 nucleotides in length, in contrast to other ncRNAs, which are usually shorter [2,3]. lncRNAs are less comparable between species; protein-coding genes tend to be homologous between mammals [4]. Four main mechanisms are involved in their expression and they are sometimes defined as *archetypes*: signaling, decoying, scaffolding and guidance [5,6]. lncRNAs seem to have an importance in physiological regulation, and their dysfunction has been implicated in cardiovascular and neurodegenerative diseases as well as in cancers [7,8]. lncRNAs seem to be important modulators in biological mechanisms and significant cofactors of tumorogenesis. lncRNA gene polymorphisms have been linked to cancer risk [9]. Systematic reviews on the association between lncRNA single-nucleotide polymorphisms (SNPs) and the overall risk of developing a cancer are starting to appear in the literature. A total of 17 SNPs in four common lncRNA genes have already been described by Zhi et al. in a meta-analysis [9]. However, the involvement of lncRNAs in endometrioid endometrial adenocarcinoma (EEC) remains to be fully addressed. Indeed, RNAs are expressed in different ways in the normal, hyperplastic and dysplastic endometrium [6]; hence, interference in RNA expression could actually lead to the clinical control of neoplastic transformation and disease progression. We offer an overview of the current knowledge of the relationship between ncRNAs and endometrial adenocarcinoma (EC) that shows promise in the diagnosis and treatment of this condition.

## 2. Endometrial Cancer: Overview

With over 189,000 new cases and about 45,000 deaths worldwide per year, EC is the second most common gynecologic tumor [10,11]. About 54,870 cases were diagnosed in the United States in 2015, with about 10,170 deaths [12]. A relevant role in EC etiology is played by social and cultural variables, as well as socio-economic disparities among ethnic groups in some countries, because of the differential availability of medical treatments. 

Two different types of EC can be identified using pathological and demographic parameters: Type I EC, also known as EEC, derives from the hyperplastic endometrium due to high endogenous estrogen production. Thus, it affects women both prior to and after menopause, and shows an average lower age of incidence (40–50 years). These tumors are usually well-differentiated, with average recurrence rates of 20%. Type II EC is post-menopausal, so the average age of incidence is about 60 years; no precise relationship with host estrogen production has been demonstrated [13]. 

About 80% of type I tumors show downregulation or mutations of the phosphatase and tensin (*PTEN*) gene [14,15], leading to an enhanced activity of the phosphatidylinositide 3-kinase–Akt–mammalian target of Rapamycin (PI3K/Akt/mTOR) signaling pathway. Furthermore, the activity of the *K-ras* oncogene or of the fibroblast growth factor receptor (FGFR) seems to lead to high levels of mitogen-activated protein kinases (MAPKs), with added phosphorylation of the estrogen receptor (ER) in a β-catenin-dependent manner [16]. This leads to unwarranted transcriptional effects as well as ER activation with a deregulation of cellular proliferation and consequent tumorigenesis [17].

Type II EC, or non-endometrioid endometrial carcinoma (NEEC), is not related to circulating estrogen levels. These tumors originate from atrophic endometrial tissue in post-menopause [18]. Type II EC can be undifferentiated or show histological features consistent with serous, endometrioid, or clear-cell differentiation. They are usually diagnosed at an advanced stage, with distant metastases already present, and their prognosis is poor even with advanced treatment [19]. More than half of Type II EC recurs within five years of surgery [13]. At proteomics, NEEC tissue samples show a high expression of mutant tumor protein 53 (TP53) and tumor suppressor protein 16 (P16), usually considered non-functional proteins [6,20].

Because protein expression in EC seems altered, protein coding regulation anomalies typically associated with RNA inhibitory activity have often been suggested. The ncRNAs show promise as diagnostic biomarkers, indicators of progression, lymph node status, therapy response, and even as targets for tumorigenesis and progression mechanisms interference with considerable therapeutic potential. ncRNA significance has been investigated in relation to endometrial cancer, providing preliminary results.

### Long Non-Coding RNAs: General Introduction

lncRNAs cover a large part of the non-coding information of the human DNA, which represents more than 90% of the whole genome. They constitute a wide and complex group of molecules with more than 200 nucleotides, usually lack an open reading frame, and in various ways are involved in the pathophysiology of cancer [2,3,5]. Their roles in the regulation of gene expression, imprinting, transcription, and post-translational processing have been documented in several types of cancer. Thousands of long intergenic ncRNAs (lincRNAs) have been identified in various mammals in genome sequencing studies. Some of these RNAs have been conserved during the evolution of various species and might have a fundamental importance in the regulation of cell mechanisms [2,3,4,5].

## 3. Competing Endogenous RNAs, miRNA, lncRNA Profiling Regulation and Cancer

The miRNAs are a class of small ncRNAs that have been associated with several diseases, including cancer [21]. They bind to target mRNAs via sequence complementarity, leading to translation inhibition and mRNA destabilization. Several lncRNAs are capable of binding to miRNAs interfering with this mechanism, thus blocking their action on target mRNAs. These lncRNAs are called ceRNAs, and several examples having a role in tumorigenesis and evolution have been published. Many lncRNA–miRNA interactions have been reported or suggested recently [22]. For example, the lncRNA named MEG3 was recently implicated as an influencer of the signal transducer and activator of transcription 3 (STAT3) expression, by altering miR-21 expression in ovarian cancer; it has been shown to function as a tumor suppressor in many cancer types [23]. Maternally expressed gene 3 (*MEG3*) is a maternally imprinted lncRNA with a tumor suppressor role in various tumors. Sun et al. [24] found significantly lower MEG3 expression in endometrial carcinoma tissues than in normal endometrial tissues. 

A cross downregulation of miRNA and lncRNAs, regulating the metastasis associated lung adenocarcinoma transcript 1 (MALAT-1) was suggested by Li et al. [25]. According to the authors, the miR-200 family members were present in a high concentration in EEC, melanoma, and some ovarian cancers. They showed that miR-200c levels were higher in most EEC specimens than in healthy tissues, while MALAT-1 levels were much lower. However, they also found that miR-200c was directly bound to MALAT-1 and miR-200c, both of which showed cross-repression, while TGF- β increased MALAT-1 expression, working as an inhibitor of miR-200c. The interruption of the repressive effect of miR-200c over MALAT-1 decreased the invasive capacity of EEC cells; the epithelial to mesenchymal cell transition (EMT) markers’ expression was altered, at least in vitro [25]. However, the abnormal expression of the lncRNA MALAT-1 or miR-200 family members were shown to facilitate EMT in various human cancers. The details of the regulatory mechanism of MALAT1 and miR-200 remain largely unknown. 

lncRNAs can be a precursor for miRNAs. For example, H19 is the gene precursor of a ubiquitous lncRNAs that show a negative or limiting regulation regarding body weight and cell proliferation. H19 is the RNA precursor of miR-675 [26]. Other miRNAs have been associated with tumor progression: Vennin et al. showed that miR-675 enhanced tumor initiation, progression, and metastasis of breast cancer cells by downregulating the *c-Cbl* and *Cbl-b* gene coding for the CBL protein [26].

This protein is an E3 ubiquitin-protein ligase involved in cell signaling [26]. The expression pattern and biological functions of the nuclear-enriched abundant transcript 1 (NEAT1) was also related to EEC. The levels of NEAT1 were up-regulated in ECC tissues and cell lines [27]. Moreover, the lncRNA cancer susceptibility candidate 2 (CASC2) was identified as a potential tumor suppressor in EC [28].

The ovarian adenocarcinoma amplified lncRNA (OVAL) is composed of three exons of 1489 nucleotides in length. OVAL was thought to encode various expressed sequence tags (ESTs) and mRNAs, but recently an alternative first exon isoform has been identified, leading to the transcription of the lncRNA OVAL [29]. The OVAL is over-expressed in Type I EC and in serous ovarian cancer (OC). Type II EC, on the other hand, is four times less likely to show upregulation of the *OVAL* gene; p53-regulated genes are upregulated in EC tissue samples with high expression of OVAL [29,30].

The carcinogenetic effect of estrogens in type I EC is well-documented [31]. Endometrial cancer and breast cancer (BC) show a similar sensitivity to estrogens. According to Bhan et al., estradiol induces lncRNA HOTAIR expression, as well as estrogen receptors and general transcription factors of RNA polymerase II [32]. Thus, HOTAIR is not only abnormally expressed in BC, but in EC as well [33]. According to He et al., HOTAIR upregulation was found in nearly three-quarters of EC samples (63 out of 87), only 20% of control endometria tested positive for this lncRNA (4 out of 30). Moreover, differentiated tumor samples (G3 grade) showed higher expression levels than Grade 1 samples. HOTAIR gene upregulation could be detected in about 50% of hyperplastic endometriums (5 out of 12). HOTAIR is also associated with metastatic disease. High levels of HOTAIR expression correlates with metastases and decreased patient survival in EC [34]. The lncRNA steroid receptor RNA activator (SRA) regulates gene expression induced by steroid receptors [35]. SRA is upregulated in BC and in other steroid-responsive tumor tissues, such as ovarian carcinoma [36]. In EC, high levels of SRA expression are found with no relation to histological tumor grade. In reference tissue samples of normal endometria, SRA expression is low and this may imply an early role in tumorigenesis [35]. Curiously, according to Lanz et al., SRA-transgenic mice did not develop any tumors, even with SRA over-expression [37].

The expression of lncRNA and their role in defining EEC subgroups and their clinical aggressiveness must be defined further. Xu et al. suggested that six lncRNAs may be the main regulators of endometrial carcinogenesis [38]. Xu measured a total of 172 lncRNAs and 188 mRNAs and reported that they were expressed differently in type I EC and the normal control samples [38]. Mathematical analysis with the gene ontology (GO) pathway analysis and the lncRNA and mRNA co-expression networks indicated that six lncRNAs (KIAA0087, RP11-501O2, FAM212B-AS1, LOC102723552, RP11-140I24 and RP11-600K151) could be the main regulators of endometrial carcinogenesis [38].

Another study drew a parallel between EEC and liver cancer (LC) in relation to lncRNA expression. Jiang et al. isolated 1931 expressed lncRNAs and gave them importance as to tumor initiation with a mathematical integrative analysis [39]. A clustering of lncRNA expression helped define three groups according to the authors: (i) basal-like, (ii) luminal-like and (iii) Catenin, β-1 (CTNNB1)-enriched subgroups. The basal-like subgroup had a much higher number of tumors with higher pathological grade (*p* < 0.0001), and TNM stage (*p* = 0.01); in the luminal-like subgroup, progesterone (PGR) and estrogen receptor (ESR1) genes were not downregulated as in the EEC basal-like subgroup. The lncRNA profile of the CTNNB1-enriched EEC subgroup was almost equal to that of the CTNNB1-enriched LC subgroup [39].

Liu et al. showed that the lncRNA Taurine Upregulated Gene 1 (*TUG1*) was involved in EC tumorigenesis due to the inhibition of miR-299 and miR-34a-5p [39]. The relationship between lncRNA-TUG1 and EC was studied in 104 EC specimens, and corresponding control tissues. lncRNA-TUG1 expression in cancer samples was higher than that in adjacent normal tissues. The authors were able to show that lncRNA-TUG1 helps the evolution and progression of EC through the inhibition of miR-299 and miR-34a-5p [40].

In a large study, 30,586 lncRNAs and 26,109 transcripts (fold change > 2.0) were found in 45 tested EC samples [40]. In particular, compared with normal tissues, 4010 lncRNA were upregulated, and 3350 of them were downregulated. Among these lncRNAs, 3 were upregulated and 4 were downregulated. Pathway analysis revealed that 24 pathways were correlated to the upregulated transcripts [41]. Chen et al. showed that miR-93 was expressed at high levels in EC samples compared to normal endometrial samples [42].

Zhai et al. looked into the expression profiles of lncRNAs and coding genes in three paired EC and adjacent healthy tissues [43]. There was significant a difference in lncRNA and coding gene expression between EC and their adjacent healthy non-tumor tissues; 53 lncRNAs (*p* value < 0.05) were differently expressed in EC, compared with controls. ASLNC04080 was the most upregulated lncRNA in 22 out of 24 samples and in HEC-1-B cell line [43]. Li et al. measured the levels of 26 lincRNAs in 176 pairs of EC and adjacent healthy biopsy samples in two separate regional Chinese populations [44]. They found that a lincRNA, LINC00672, was abnormally downregulated during the development of EC. LINC00672 is a p53-targeting lincRNA, acting along with heterogeneous nuclear ribonucleoproteins as a suppressive cofactor that reinforces p53-mediated suppression of the LIM and SH3 domain protein (LASP)-1; this mechanism could possibly be associated with increased tumor aggressiveness [44].

Sun et al. looked into lncRNAs using support vector machine and random forest methods [45]. All expressed lncRNAs were ranked according to the standardized drop in probable prediction accuracy. An odds analysis for predictive performance was performed, adding one lncRNA at a time starting with the top two lncRNAs; of the ranked list, five lncRNAs were the optimal mix for diagnostic accuracy. When choosing more than five lncRNAs, there was a downward trend in predictive performance. The top five lncRNAs (FLJ27354, RP11-275I14.4, VIM-AS1, CTB-51J22.1 and RP11-229P13.20) were selected as optimal predictive lncRNA biomarkers of uterine corpus EC (UCEC) progression. Only one lncRNAs (FLJ27354) was clearly associated with disease progression, the other top four lncRNA markers (RP11-275I14.4, VIM-AS1, CTB-51J22.1 and RP11-229P13.20) were silent. Using these top five markers led to a high discriminatory performance in distinguishing advanced stages from early stages with 78% prediction accuracy, 96.6% sensitivity and 76.6% specificity. These lncRNAs could have a functional role in the progression of EC initiating, and in the promotion of important cancer-related processes [45].

## 4. Regulation of Cell Growth by Long Non-Coding RNAs in Endometrioid Endometrial Adenocarcinoma

lcnRNA H19 levels were significantly higher in EC tissues than in para-tumoral samples [45]. The expression pattern of H19 in EC tissues was addressed by quantitative polymerase chain reaction (Q-PCR), and its function characterized in the EC cell line through the inhibition of expression with small interfering RNAs (siRNAs) [45]. Lowering H19 levels did not affect the growth of HEC-1-B EC cells, but it did suppress their capability to migrate and invade. Furthermore, H19 downregulation decreased the transcription factor Snail, associated with E-cadherin downregulation, destabilization of the adherent junction, and cellular polarization, and increased matrix metalloproteinase (MMP) expression, with no effect on vimentin levels, indicating at least a partial reversion of EMT [46]. H19 was able to enhance EC aggressiveness by modulating the EMT process [47]. 

The lncRNA BANCR promotes EC cell replication and tumor invasion, by regulating Matrix MetalloProteinase-1 (MMP1) and MMP2, and via extracellular signal-regulated kinase-1 (ERK)–MAPK signaling pathways. BANCR was highly expressed in type 1 EC tissues, promoting EC cell proliferation, migration, and invasion by activating the ERK–MAPK signaling pathway that regulates MMP2 and MMP1 expressions [48]. For this reason, BANCR could become a prognostic marker and important drug target in type 1 EC. MEG3 and two signaling molecules, Notch1 and Hes1, were detected in both EC tissues and cell lines by RT-PCR and western blot analysis, all of them showing significant expression. Lentiviral vectors with whole MEG3 transcripts or Short (or small) hairpin RNA (shRNA) targeting MEG3 (shMEG3) was transinfected to evaluate cell proliferation. MEG3 dysregulation was studied in xenograft models (established by subcutaneous implantation), and tumor growth was compared. Important downregulation of MEG3 was observed in EC samples compared to controls, while levels of Notch1 and Hes1 were found to be significantly upregulated. Cell proliferation was greatly inhibited by MEG3 over-expression, while the opposite was observed in MEG3 knockout cells. Interestingly enough, MEG3 changes could be reversed by Notch1 regulators. Moreover, over-expression of MEG3 was strongly associated with repressed in vivo growth, along with Notch signaling inhibition. Downregulated MEG3 was an important anti-proliferative factor in EC, by repressing the Notch signaling pathway [49].

Cisplatin-resistant Ishikawa cells (a human EC cell line) showed high autophagy activity compared with non-cisplatin-resistant parent Ishikawa cells. After lncRNA profiling, HOTAIR was identified as the culprit: it was downregulated both in cisplatin-resistant Ishikawa cells and parental Ishikawa cells treated with cisplatin. RNA interference on HOTAIR reduced the proliferation of cisplatin-resistant Ishikawa cells and increased autophagy in cisplatin-resistant Ishikawa cells with or without cisplatin treatment. Furthermore, beclin-1, multidrug resistance (MDR), and P-glycoprotein (P-gp) expression was mediated by lncRNA HOTAIR. It was concluded that HOTAIR can regulate the cisplatin resistance of human EC cells, regulating autophagy by influencing Beclin-1, MDR, and P-gp expression [30].

The tumor suppressor candidate 7 (TUSC7) is an antisense lncRNA: if downregulated, it acts as a possible tumor suppressor in several cancers. In the study by Shang et al., the low expression of TUSC7 was associated with high pathological stages of EC, which revealed that TUSC7 might be involved in the initiation and progression of EC. Moreover, the expression of TUSC7 in EC samples and EC cell lines resistant to cisplatin CDDP, and Taxol was lower than that in sensitive EC tissues and cell lines, which indicated that the TUSC7 expression level was positively related to the response of EC patients to chemotherapy with CDDP and Taxol [50].

Li et al. showed that the expression and function of NEAT1 in EEC was elevated in ECC samples and cell lines, with a high expression levels of NEAT1 being associated with enhanced cell growth, colony formation ability, and invasive and migratory ability in pGCMV-NEAT1, while limiting the expression of NEAT1 in HEC-59 cells by siNEAT1 transfection showed the opposite effects [27]. Guo et al. demonstrated that the growth arrest-specific 5 (GAS5) functions as a significant tumor suppressing lncRNA in EC. Inhibiting the expression of miR-103, GAS5 enhances the expression of the *PTEN* gene leading to cancer cell apoptosis. GAS5 downregulation could represent an important factor in the pathogenesis of EC [51].

Moreover, the lncRNA Fer-1-like protein 4 (*FER1L4*) might suppress EC cell proliferation. A plasmid containing FER1L4 was transfected into HEC-50 cells, showing low levels of FER1L expression. Western blot analysis was used to determine PTEN expression and Akt phosphorylation. FER1L4 was significantly downregulated in EC tissues compared to controls. This was positively correlated with decreased *PTEN* expression. Therefore, FER1L4 could promote PTEN expression and inhibit Akt phosphorylation. A significant decrease of cell proliferation was observed in FER1L4 overexpressing cells, with cell cycle arrest at G0/G1 phase and enhanced apoptosis [52].

## 5. Long Intergenic Non-Protein Coding RNA, Regulator of Reprogramming and Endometrioid Endometrial Adenocarcinoma

Ample evidence indicates that linc- Regulator of Reprogramming (ROR) has a significant role in tumor initiation and progression. In tumorigenesis, linc-ROR might act as an oncogene [53]. In general, linc-ROR can be easily associated with cell proliferation, differentiation, apoptosis, invasion, and metastasis in many human cancers. However, the molecules that act as mechanisms that mediate linc-ROR’s effects must still be clearly identified before linc-ROR can be used in tumor treatment. Many studies have shown the importance of linc-ROR as a tumor marker. Marked upregulation of linc-ROR can be observed in various cancers, like BC [54,55], pancreatic cancer (PC) [56,57], hepatocellular cancer (LC) [58,59], EC [60,61], and nasopharyngeal carcinoma [62]; furthermore, linc-ROR works as a tumor suppressor in glioma [53]. Although linc-ROR is over-expressed in various cancers, the target genes are variable and depend on the cell type.

In the paper by Chen et al., RNA was isolated from malignant and adjacent non-affected endometrial tissue from six patients with low-grade, type I EC. Subsequently, Illumina paired-end RNA sequencing was performed, to determine differential transcriptome expression patterns. Linc RNAs were specifically analyzed. LINC00958 was upregulated, and four lincRNAs including LINC01480, LINC00645, LINC00891 and LINC00702 demonstrated exquisite specificity for malignant endometrium compared to normal endometrium, while also distinguishing EC from OC and Cervical Cancers (CC) [62].

Zhou et al. studied a linc-RoR which was possibly implicated in the expression regulation of core stem cell transcription factors (TFs) as ceRNA [60]. Adenovirus vectors carrying green fluorescent protein (GFP) were transfected into E-Twenty-six (ETs) TFs to evaluate the effect of down- or upregulation of miR-145, linc-RoR or Dicer. Expression of linc-RoR and core stem cell TFs was associated with cell differentiation of the ET TF, whereas miR-145 expression increased after ET differentiation. A higher expression of miR-145 successfully led to the downregulation of linc-RoR and core TFs. The opposite was achieved by knocking down miR-145 expression. The effects of miR-145 could be nullified by increasing the expression of linc-RoR in ETs or mutated targeted sequences in linc-RoR. Reduced Dicer expression were able to enhance the expression of linc-RoR and core TFs. The linc-RoR acted as a miR-145 inhibitor on the differentiation of ETs in endometrial carcinogenesis [60].

## 6. Micro IRNA in Endometrioid Endometrial Adenocarcinoma

MicroRNA-93 is derived from a paralog (miR-106b-25) of the miR-17-92 cluster and has been implicated in tumor initiation and progression of breast, colorectal, hepatic, lung, ovarian, and pancreatic cancer.

The article by Chen et al. demonstrated miR-93 overexpression in EC tissue samples as compared with normal endometrium. According to Chen et al., miR-93 overexpression promotes cell migration and invasion, by downregulation of E-cadherin while N-cadherin expression in EC cells seems significantly enhanced [42].

miRNA-205 is probably a key regulator of EC gene expression. Higher levels of miRNA-205 expression were measured in EC with invasion limited to less than half of the myometrium and initial EC. A better survival rate was associated with higher levels of miRNA-205 using Kaplan–Maier analysis (*p* = 0.034); miRNA-205 could thus be used as a prognostic marker [63]. Levels of miR-944 were analyzed in 68 cancerous and 20 normal endometrial samples: miR-944 was highly overexpressed in EC tissues compared to normal controls. Furthermore, increased levels of miR-944 were observed in EC cell lines. miR-944 expression was associated with the EC stages and pathology results of the International Federation of Gynecology and Obstetrics (FIGO) [64].

Using small-RNA sequencing and microarrays, significant differences in Small Non-Coding RNAs (sncRNA) expression patterns between normal, hyperplastic and neoplastic endometrium could be easily identified. The authors propose a sncRNA signature (129 microRNAs, 2 of which were previously unknown, 10 piRNAs and 3 snoRNAs) of neoplastic transformation. This new sncRNA signature probably reflects events leading to EC development [65,66].

### Micro RNA as a Marker and Promoter of Tumor Aggression in EEC

Can miRNA evaluation accurately predict the risk of lymph node extension in EC patients? Eighty-six matched EC cases evenly distributed between lymph node-positive and lymph node-negative cases, were analyzed in the study by Ahsen et al. [67]. A predictive matrix was generated using genomic miRNA expression in EC node-positive patients. An independent set of 28 other tumor samples was similarly characterized and was used in a test cohort. A predictive signature of miRNA expression was generated and used to predict the metastatic status of the independent test cohort. Using 18 miRNAs, 100% accuracy was obtained with the control group. The EC group showed 90% accuracy in the node-positive cases, and 80% in node-negative cases (false discovery rate, FDR = 6.25%) [67]. The Dicer1 endoribonuclease plays a critical role in miRNA biogenesis. Bahubeshi et al. showed that Dicer1 dysfunction leads to the enrichment of tumor stemness and tumor aggression both in vitro and in vivo [68]. Loss of Dicer1-induced abnormal expression of the miRNA let-7 family, which comprises well-known tumor suppressors, thus regulating stemness in EC cells [68].

Hu showed that silencing nc886, a ncRNA, leads to apoptosis of human EC cells in vitro. In addition, tests results showed higher level of nc886 in the late phase of human EC tissue, slightly more than in the early phase of EC but always much higher than in normal endometrial tissue. nc886 inhibition led to increased protein levels of phosphorylated PKR (p-PKR) and caspase-3, NF-kB and vascular endothelial growth factor (VEGF) were on the opposite decreased. The rate of apoptosis in the nc886 inhibition group was increased and cell proliferation was much slower as compared to controls [69].

The inhibition of the enhancer of zeste homolog 2 (EZH2), (a histone-lysine N-methyltransferase enzyme), and the reactivation of tumor suppressor miRNAs might lead to a functional cancer therapy regime strategy. Ihira et al. observed that EZH2-suppressed miRNA let 7b and miR-361, two possible tumor suppressors, with consequent EC cell proliferation and invasion, and negated their stem-cell-like properties. In EC cells, EZH2 induced and worked together with the transcription factor Yin Yang 1 (YY1) to suppress miR-361, which in turn upregulated Twist, a target of miR-361. Treating EC cells with GSK343, a targeted EZH2 inhibitor, had the same effect of a siRNA-mediated EZH2 knockdown, upregulating miR-361 and downregulating Twist expression. Combining GSK343 with 5-AZA-2’-deoxycytidine suppressed cell proliferation with an apparent synergic effect and blocked invasion in vitro, lowering the tumor size and weight in EC cell grafted mice. Worse patient outcomes could be predicted: a quantitative real-time PCR (Q-RT-PCR) analysis of 24 primary EC tissues showed that lower let-7b and miR-361 levels were dire prognostic indicators. Results were confirmed on a large patient data set from The Cancer Genome Atlas [70]. EZH2 might drive EC progression by regulating miR-361–Twist signaling and indicate EZH2 inhibition as a possible anti-EC therapeutic strategy [68]. miRNA-93 overexpression increased cell migration and invasion, and downregulated E-cadherin expression while increasing N-cadherin expression [42]. Chen et al. showed, by dual-luciferase reporter assay, that miR-93 directly binds to t 30 untranslated region of Forkhead Box A1 (FOXA1), and that its over-expression downregulated FOXA1 expression. On the other hand, miR-93 inhibitor transfection upregulated FOXA1 expression at protein and mRNA levels. The conclusion was that miR-93 may aid or initiate the process of EMT in EC cells by targeting FOXA1 [42]. An intriguing hypothesis by Gong et al. was that the miRNA-194 downregulation (miRNA-194, is an inhibitor of EMT in several cancers) is associated to a worse prognosis in human EC. Self-renewal factor Sox3 induces EMT at gastrulation and is also involved in EMT in several cancers. Ectopic expression of microRNA-194 in EC stem cells induced a MET by normalizing E-cadherin expression, decreasing vimentin expression, and interfering with cell invasion in vitro. Moreover, overexpression of microRNA-194 inhibited EC stem cell invasion or metastasis in vivo by the injection of adenovirus microRNA-194. This demonstrates the new mechanism by which Sox3 contributes to EC stem cell invasion and suggests that repression of Sox3 by miRNA-194 may have a therapeutic use in the suppression of EC metastasis. The cancer stem cell marker CD133 might be the most significant surface marker of EC stem cell [69]. The effects of miR-29b regulate via MAPK–ERK and PI3K–Akt signaling pathways the angiogenesis in EC by targeting Vascular endothelial growth factor A (VEGFA). In the paper by Chen et al., EC tissues were compared with the adjacent normal tissues and showed that miR-29b expression was downregulated, while the mRNA and protein expression of VEGFA, ERK, Akt, mTOR and B-cell lymphoma 2 (Bcl-2) were upregulated [71]. The left-right determination factor 2 (LEFTY2), considered a suppressor of cell proliferation, tumor growth, and regulator of stem cell properties and embryonic differentiation, seems to be a negative regulator of cancer cell programming. 

LEFTY2 seems to downregulate MKi67 expression and focal adhesion kinase (FAK) activity, while upregulating miR-200 and E-cadherin. It is thus a significant negative regulator of endometrial cell growth and migration, probably under-expressed in EEC [72].

## 7. Conclusions

**Figure 1:**
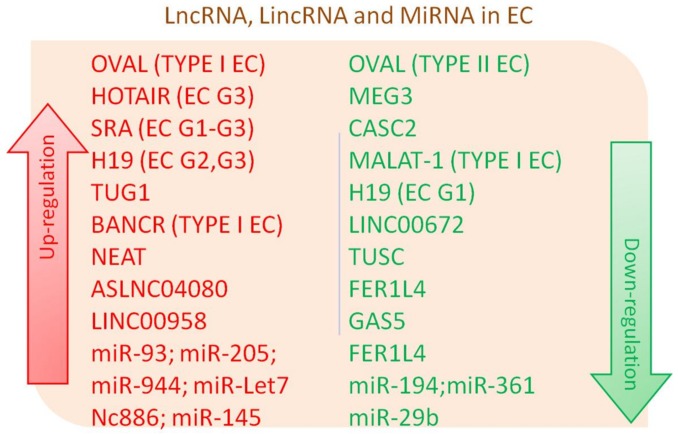
Differentially expressed long non-coding RNA (lncRNA), long intergenic non-coding RNAs (lincRNAs) lincRNA and micro RNAs (miRNA) in Endometrial Cancer (EC).

Abnormal expression of several sets of genes is probably the prime mover or a significant factor in the progression of a cancer, and both mechanisms can be at work in sequence. Genetic alterations include both mutational and epigenetic changes, such as methylation, acetylation, and phosphorylation of nuclear chromatin [73,74,75]. Cellular genes are in charge of proliferation, apoptosis, and stem cell differentiation; all of them can undergo epigenetic modifications in cancer [76,77]. The significance of ncRNA is starting to be put under investigation, but more questions than answers are available for now. Is ncRNA a possible marker of cancer risk? Can it be used as a biomarker for tumor diagnosis and as an indicator of tumor progression or of a risk of tumor metastases? Can selective activation or inhibition of RNA pathways help cure or control the aggressiveness of the disease? Are results valid for all cancers? Do some of the promising preliminary results obtained in other conditions apply to EC? Various information-carrying genes have shown important biological and regulatory functions without expressing a translation of their information, such as classically the production of proteins. In particular, lncRNAs show specific and altered expression patterns in EC compared to normal endometrial tissue (Table 1, Figure 1). Some well-known factors associated with EEC show an association with ncRNA expression. Levels of sex hormones are involved in the development of EC. Hormones affect RNAs like lncRNAs SRA, H19 and HOTAIR, and their levels could play a role in the carcinogenesis of EC. Estradiol is an important link between expression of these lncRNAs as it correlates positively with upregulation of H19 and HOTAIR in EC [32,78]. Preliminary in vitro studies showing the reduced growth of EEC cells indicate that a possible new therapeutic pathway for EEC is possible. Study of the mechanisms of expression of RNA in EEC should be pursued, and their effect on cancer growth clarified. lncRNAs could be useful clinical biomarkers of EEC for progression to advanced stages. Hence, lncRNAs could be optimal biomarkers, useful to identify patients at high risk for progression to advanced stage, and the profiling of lncRNAs could actually offer improved diagnostic accuracy.

## Figures and Tables

**Table 1:** Non-coding RNA expression and EC.

NcRNA	Type of ncRNA Regulation/Interaction	Type of EC Regulation/Pathway Interaction	In Vivo or In Vitro Assay or Humans	Reference
LncRNA OVAL	down and upregulation	downregulation in type II EC and upregulation in type I EC	In vitro assay from EC tissue	[6]
LncRNA MALAT-1	downregulation/interaction with miR-200	downregulation in EC through miR-200	In vitro assay from EC tissue, xenograft tumor model	[25]
LncRNA TUG1	interaction with miR-299/miR-34a-5p	promotion of EC via miR-299 and miR-34a-5p inhibition	In vivo mouse assay, in vitro assay from EC tissue and HEC-1-A cell lines	[40]
LncRNAs HOTAIR	upregulation	downregulation in Cisplatin-Resistant Ishikawa Cells (CRIC) and parental IC treated with cisplatin; up-regulation with EC tumor grade increase	In vitro assay CRIC cells; in vitro assay from EC tissue	[30,34]
LncRNAs H19	upregulation	upregulation with EC tumor grade increase and other features associated with poor prognosis	In vitro assay from EC tissues and EC cell line	[46]
LncRNAs SRA	upregulation	upregulation regardless of histological tumor grade	In vitro assay from EC tissues	[36]
LncRNA BANCR	upregulation	promotion of EE cell proliferation and invasion by MMP2 and MMP1 regulation via ERK–MAPK signaling pathway in type 1 EC	In vitro assay from EC tissues	[48]
LncRNAs panel (e.g., KIAA0087, RP11-501O2, FAM212B-AS1, LOC102723552, RP11-140I24 and RP11-600K151)	differential regulation/interaction with 188 mRNAs	differential expression in type I EC	In vitro assay from EC tissues	[38]
LncRNA CASC2	downregulation	potential role as a tumor suppressor	In vitro assay from EC tissues	[29]
LncRNA ASLNC04080	upregulation	upregulation in 22–24 EC tissues and HEC-1-B cell line	In vitro assay from EC tissues and EC cell line	[43]
LncRNAs panel (e.g., FLJ27354, RP11-275I14.4, VIM-AS1, CTB-51J22.1 and RP11-229P13.20)	up and downregulation	potential biomarkers of Uterine Corpus (UCEC)	In vitro assay from EC tissues	[45]
LncRNA TUSC7	downregulation	related to EC tumorigenesis and progression	In vitro assay from EC tissues	[50]
LncRNA FER1L4	downregulation	decrease of cell proliferation in FER1L4-overexpressing cells	In vitro assay from EC cell line	[52]
LncRNA NEAT	upregulation	increase of Nuclear Enriched Abundant Transcript 1 (NEAT1) in EC	In vitro assay from EC tissues and cell lines	[27]
LncRNA MEG3	downregulation	anti-proliferative role in EC by repressing Notch signaling pathway	In vitro assay from EC tissues and cell lines	[49]
LncRNA GAS5/miR-103	downregulation / inhibition of miRNA-103 expression	tumor suppressor	In vitro assay from EC tissues and cell lines	[51]
LincRNA LINC00958	upregulation	increase of LINC00958 in EC	In vitro assay from EC tissues	[62]
LincRNA LINC00672	downregulation	downregulation during EC development	In vitro assay from EC tissues	[44]
NcRNA Nc886	upregulation	upregulation of nc886 in EC late phases, compared to early stages and Normal Endometrial Tissues (NET)	In vitro assay from EC tissues	[68]
miRNA-93	upregulation	over-expression associated to cell migration and invasion	In vitro assay from EC tissues and cell lines	[42]
miRNA-205	upregulation	prognostic marker associated with better overall survival	In vitro assay from EC tissues	[63]
miRNA-944	upregulation	over-expression in EC tissues compared to NET	In vitro assay from EC tissues	[65]
miRNA-29b	miRNA-29b/MAPK/ERK and PI3K/Akt signaling pathways	inhibition of angiogenesis by targeting VEGFA through MAPK/ERK and PI3K/Akt signaling	In vitro assay from EC tissues and cell lines	[73]
miRNA-145	upregulation	upregulation of miR-145 lead to downregulation of linc-RoR7Dicer	In vitro assay from EC tissues	[60]
miRNA-361	miR-361/twist signaling and miR-361/let-7b downregulation	lower let-7b, twist signaling and miR-361 are associated with worse patient outcome	In vitro assay from EC tissues	[71]
miRNA-194	downregulation	downregulation associated to EC poor prognosis	In vitro assay from EC tissues and cell lines	[73]
